# Influence of Anode Potentials on Current Generation and Extracellular Electron Transfer Paths of *Geobacter* Species

**DOI:** 10.3390/ijms18010108

**Published:** 2017-01-06

**Authors:** Souichiro Kato

**Affiliations:** 1 Bioproduction Research Institute, National Institute of Advanced Industrial Science and Technology (AIST), 2-17-2-1 Tsukisamu-Higashi, Toyohira-ku, Sapporo, Hokkaido 062-8517, Japan; s.katou@aist.go.jp; Tel.: +81-11-857-8968; 2 Division of Applied Bioscience, Graduate School of Agriculture, Hokkaido University, Kita-9 Nishi-9, Kita-ku, Sapporo, Hokkaido 060-8589, Japan

**Keywords:** *Geobacter*, current generation, anode potential, electrochemistry, microbial fuel cells

## Abstract

*Geobacter* species are capable of utilizing solid-state compounds, including anodic electrodes, as electron acceptors of respiration via extracellular electron transfer (EET) and have attracted considerable attention for their crucial role as biocatalysts of bioelectrochemical systems (BES’s). Recent studies disclosed that anode potentials affect power output and anodic microbial communities, including selection of dominant *Geobacter* species, in various BES’s. However, the details in current-generating properties and responses to anode potentials have been investigated only for a model species, namely *Geobacter sulfurreducens*. In this study, the effects of anode potentials on the current generation and the EET paths were investigated by cultivating six *Geobacter* species with different anode potentials, followed by electrochemical analyses. The electrochemical cultivation demonstrated that the *G. metallireducens* clade species (*G. sulfurreducens* and *G. metallireducens*) constantly generate high current densities at a wide range of anode potentials (≥−0.3 or −0.2 V vs. Ag/AgCl), while the subsurface clades species (*G. daltonii*, *G. bemidjensis*, *G. chapellei*, and *G. pelophilus*) generate a relatively large current only at limited potential regions (−0.1 to −0.3 V vs. Ag/AgCl). The linear sweep voltammetry analyses indicated that the *G. metallireducens* clade species utilize only one EET path irrespective of the anode potentials, while the subsurface clades species utilize multiple EET paths, which can be optimized depending on the anode potentials. These results clearly demonstrate that the response features to anode potentials are divergent among species (or clades) of *Geobacter*.

## 1. Introduction

The genus *Geobacter* is a member of the Deltaproteobacteria and can utilize solid-state compounds such as iron and manganese oxides as the electron acceptors of respiration [1]. The ability to utilize solid compounds as electron acceptors is referred to as extracellular electron transfer (EET) [2]. The molecular mechanisms of EET have been extensively investigated on the model species, *Geobacter sulfurreducens*, and the outer membrane *c*-type cytochromes (OMCs) were found to play a crucial role in this process [3,4]. In addition to naturally occurring metal compounds, some *Geobacter* species have abilities to utilize artificial conductive materials, including graphite and metal electrodes, as the electron acceptors [5]. Consequently, *Geobacter* species have attracted considerable attention by researchers developing various bioelectrochemical systems (BES’s) such as microbial fuel cells (MFCs) [6,7,8].

MFCs are one of the most extensively investigated BES’s, in which oxidation of organic substrates and current production (anodic reactions) by EET-harboring microorganisms are coupled with a current consuming reaction (cathodic reactions, generally reduction of O_2_) [9]. Although MFCs have been anticipated as a novel technology for low-energy intensive wastewater treatment systems, further advancements, especially improvements of power outputs, are required for commercialization [7]. Recent studies disclosed that the power output of MFCs is affected by diverse factors, including substrate types and amounts, the materials and forms of electrodes, and the reactor configurations [7]. Among them, the effects of anode potentials have recently attracted considerable attention. Anode potential in practical MFCs is controllable by changing an external resistance and/or the ratio of the anode/cathode area. Previous studies demonstrated that the power output of MFCs can be improved by simply tuning anode potentials (or external resistances in some cases) [10,11,12,13,14,15,16,17,18,19]. Furthermore, most of these studies pointed out that the anode potentials also have an influence on the microbial community structures on electrodes [12,13,14,15,16,17,18,19].

The effects of anode potentials on the physiologies of current-generating microorganisms have also been investigated using several model species such as *G. sulfurreducens* and *Shewanella oneidensis*. Previous studies have demonstrated that anode potentials affect current generation properties, EET paths, and the thickness of biofilm generation of *G. sulfurreducens* [20,21,22,23] and *S. oneidensis* [24,25,26]. Furthermore, recent studies revealed that gene expression patterns, metabolic pathways, and cellular redox status are also under the control of anode potentials [27,28,29,30]. However, since all these studies are intended for the restricted model species, knowledge on current-generating properties and responses to anode potentials of non-model microorganisms is quite limited.

So far, more than 20 species have been described in the genus *Geobacter* and are grouped into three clades, namely the *G. metallireducens* clade (including *G. sulfurreducens* and *G. metallireducens*), the subsurface clade I (including *G. daltonii* and *G. bemidjensis*), and the subsurface clade II (including *G. chapellei* and *G. pelophilus*) [31]. Although all of the *Geobacter* species so far described are capable of reducing solid-state metal oxides, details on the EET abilities and responses to anode potentials have been investigated only for the model species, *G. sulfurreducens* [20,21,22,23,27,29,30]. However, non-model *Geobacter* species in the subsurface clades have often been detected from various BES’s as dominant current generators [18,32,33]. Furthermore, some previous studies showed that a simple modification of the anode potentials often results in several different *Geobacter* species dominating as the major current generators [17,18,19], while the mechanisms for selection of certain *Geobacter* species are unknown.

In this study, EET abilities and responses to anode potentials were investigated by cultivating six *Geobacter* species in electrochemical cells with different anode potentials, followed by electrochemical analyses. These experiments clearly demonstrated that the effects of anode potentials on current generation profiles and the switching of EET paths are highly divergent among the various *Geobacter* species.

## 2. Results and Discussions

### 2.1. The Effects of Anode Potentials on Current Generation by Geobacter Species

**Figure 1:**
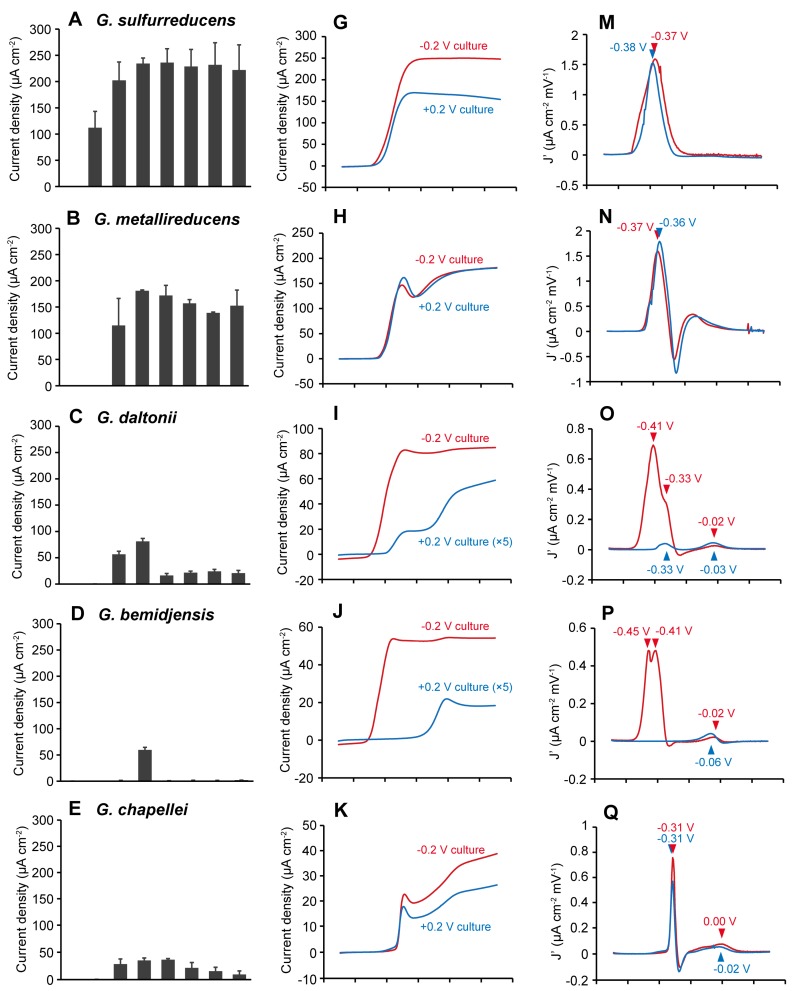

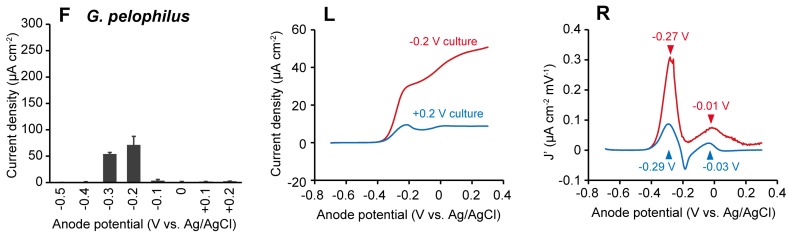
The effects of anode potentials on six *Geobacter* species. (**A**–**F**) The maximum current densities at each anode potentials. Data are presented as the means of three independent cultures and error bars represent standard deviations. (**G**–**L**) The linear sweep voltammetry (LSV) patterns (scan rate of 5 mV·s^−1^) of six *Geobacter* species cultivated at −0.2 (red) and +0.2 (blue) V vs. Ag/AgCl. In (**I**,**J**) the current densities of +0.2 V cultures are enlarged (×5). (**M**–**R**) The first derivative of the LSV data shown in (**G**–**L**). Arrowheads indicate peak potentials.

In order to investigate the effects of anode potentials on current generation properties, six *Geobacter* species (*G. sulfurreducens*, *G. metallireducens*, *G. daltonii*, *G. bemidjensis*, *G. chapellei*, and *G. pelophilus*) were cultivated in three-electrode electrochemical cells with acetate (10 mM) as the electron donor and a tin-doped indium oxide (ITO) glass electrode poised at different potentials (−0.5 to +0.2 V vs. Ag/AgCl) as the electron acceptor. The time course of current production and the maximum current densities (current per unit of electrode surface area) generated by each *Geobacter* species grown with different anode potentials are shown in Figure S1 and Figure 1A–F, respectively.

*G. sulfurreducens* and *G. metallireducens* are members of the *G. metallireducens* clade [31]. These species generated a significantly larger current than the other species (Figure 1A–F). The current generation patterns of these species were quite similar and were not affected by changes in anode potentials ranging from −0.2 to +0.2 V (Figure 1A,B). *G. sulfurreducens* generated a current at a potential range of −0.4 to +0.2 V. The current densities at −0.3 to +0.2 V were almost constant (202 to 236 μA·cm^−2^), while those at −0.4 V were significantly lower (ca. 112 μA·cm^−2^). *G. metallireducens* generated a current in a potential range of −0.3 to +0.2 V, and the current densities at −0.2 to +0.2 V were nearly constant (138 to 181 μA·cm^−2^). The current densities at −0.3 V (ca. 115 μA·cm^−2^) were significantly lower than those at −0.2 to +0.2 V. Such high current generation abilities have previously been observed for the *G. metallireducens* clade species [34,35]. Furthermore, similar flexibility in potential ranges necessary for current generation have been reported for *G. sulfurreducens* [21,23].

By contrast, other *Geobacter* species showed significant dependence on a narrow range of potential in current generation properties. *G. daltonii* (subsurface clade I) generated relatively large currents at −0.3 and −0.2 V (56 to 81 μA·cm^−2^), while the current densities at more positive potential regions were significantly lower (19 to 28 μA·cm^−2^) (Figure 1C). *G. bemidjensis* (subsurface clade I) only generated significant current when the anode was poised at −0.2 V (ca. 60 μA·cm^−2^, Figure 1D), and the current was quite small at the other potentials (<2 μA·cm^−2^). *G. chapellei* (subsurface clade II) generated a relatively large current at a potential range of −0.3 to −0.1 V (28 to 37 μA·cm^−2^), while the current gradually decreased with increasing anode potential (Figure 1E). *G. pelophilus* (subsurface clade II) generated a significant current only at −0.3 and −0.2 V (54 to 72 μA·cm^−2^), and generated quite a small current at the other potentials (<3 μA·cm^−2^) (Figure 1F).

Rotaru et al. [35] investigated current generating capabilities of various *Geobacter* species and reported that the *G. metallireducens* clade species are able to generate a significant current at a positive anode potential (ca. 200 to 220 μA·cm^−2^ at +0.3 V), while *Geobacter* species in the subsurface clades are not (<80 μA·cm^−2^). Furthermore, previous articles reported that *G. bemidjensis* [35,36,37] and *G. pelophilus* [37] are unable to generate currents (at +0.2 or +0.3 V, respectively). The results in this study are consistent with these previous reports, where species in the subsurface clades showed an inferior current production at positive anode potentials. By contrast, all subsurface clade species generated a relatively large current at negative potential regions (−0.2 to −0.3 V). Importantly, this is the first report to demonstrate current production by *G. bemidjensis* and *G. pelophilus*, which were previously not thought to be current-generating bacteria [35,36,37]. So far, enrichment cultures of current-generating bacteria and confirmation of their current-generating abilities have generally been conducted under limited conditions with positive anode potentials (usually ≥+0.2 V). This study points out that enrichment cultures and the current-generation test under more negative anode potentials would be beneficial for the discovery of unidentified current-generating microorganisms.

### 2.2. The Effects of Anode Potentials on EET Paths of Geobacter Species

In order to elucidate the effects of anode potentials on EET paths, linear sweep voltammetry (LSV) analyses were conducted on cells of six *Geobacter* species cultivated at −0.2 and +0.2 V. The results of LSV analyses are shown in Figure 1G–L. Furthermore, in order to elucidate the potential windows of each EET path, first-derivative analyses on the LSV data were performed (Figure 1M–R).

**Figure 2:**
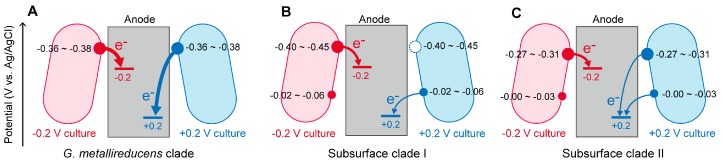
The schematic images of the switching of extracellular electron transfer (EET) paths responding to anode potentials. (**A**) The *G. metallireducens* clade species; (**B**) the subsurface clade I species; and (**C**) the subsurface clade II species. Red and blue circles represent EET paths (putatively different outer membrane *c*-type cytochromes (OMCs)). Filled and open circles represent EET paths expressed and not expressed, respectively.

The LSV patterns of the *G. metallireducens* clade species (*G. sulfurreducens* and *G. metallireducens*) cultivated at −0.2 and +0.2 V showed similar sigmoidal curves that steeply increased above ca. −0.4 V (Figure 1G,H). The first-derivative of the voltammograms showed only one major peak at ca. −0.36 to −0.38 V. These results indicate that *G. metallireducens* clade species express only one major EET path and generate a relatively large current independent of the anode potentials (Figure 2A).

By contrast, the cells of the subsurface clade I species (*G. daltonii* and *G. bemidjensis*) displayed drastically different LSV patterns between −0.2 and +0.2 V cultivations (Figure 1I,J). The first-derivative analyses indicated that *G. daltonii* have three different EET paths at voltages of ca. −0.41, −0.33, and −0.02 V (Figure 1O). The *G. daltonii* cells cultured at −0.2 V showed one major (−0.41 V) and two minor (−0.33 and −0.02 V) peaks, while those cultured at +0.2 V showed only two peaks (−0.33 and −0.03 V). Similarly, the first-derivative analyses on LSV data of *G. bemidjensis* disclosed that the −0.2 V culture shows a major peak at −0.41 to −0.45 V and a minor peak at −0.02 V, while the +0.2 V culture shows only one peak at the positive region (−0.06 V). These results indicate that the subsurface clade I species alter their EET paths depending on the anode potentials (Figure 2B); these species express multiple EET paths and generate relatively large currents via the most negative EET paths under the negative anode potential conditions, while decreasing the expression levels of the most negative EET paths under the positive anode potential conditions to generate a relatively small current via the positive EET path(s).

The LSV patterns of the subsurface clade II species (*G. chapellei* and *G. pelophilus*) cultured at −0.2 and +0.2 V were similar (Figure 1K,L). The first-derivative analyses revealed that both species display one major peak at the negative potential region (−0.27 to −0.31 V) and one minor peak at the positive potential region (0.00 to −0.03 V), independent of the potentials applied for their cultivation. These results indicate that the subsurface clade II species constitutively express two different EET paths, generate a relatively large current via the negative EET path under the negative anode potential conditions, and generate a relatively small current via both the negative and positive EET paths under the positive anode potential conditions (Figure 2C).

### 2.3. Implications

This is the first study to investigate the variations in diverse *Geobacter* species regarding the changes in current generating properties and the switching of EET paths dependent on anode potentials. Collectively, the response features to anode potentials were totally divergent among species (or clades) of *Geobacter*. The *G. metallireducens* clade species (*G. sulfurreducens* and *G. metallireducens*) appear to utilize only one EET path and generate notably large current independent of anode potentials (Figure 2A). The subsurface clade I species (*G. daltonii* and *G. bemidjensis*) appear to alter the EET paths dependent on anode potentials; negative or positive EET paths are dominantly utilized for current generation at negative or positive anode potentials, respectively (Figure 2B). In the case of the subsurface clade II species (*G. chapellei* and *G. pelophilus*), both negative and positive EET paths appear to be constitutively expressed, and the appropriate EET paths appear to contribute to current generation (Figure 2C). These variations in responses to anode potentials would be one of the important selection factors resulting in the dominance of *Geobacter* species on anodic electrodes. In this study, the molecular mechanisms of the responses of EET paths were not elucidated. It has been well known that OMCs play a crucial role in the EET process of *Geobacter* species [3,4,38]. Previous studies have reported that changes in expression patterns of multiple OMCs contribute to shifts in EET paths [19,37]. *Geobacter* species have a large number of *c*-type cytochrome genes in their genomes (>60 genes), and the variety of OMCs are somewhat different among species [39]. It is assumed that the regulatory mechanisms for the expression of OMC genes are also different among *Geobacter* species, which causes the differences in responses to anode potentials observed in this study. Further studies, including comparative genomic/transcriptomic analysis and investigation of electrochemical properties of OMCs from diverse *Geobacter* species, are required to clarify the molecular mechanisms of the EET path switching depending on anodic potentials.

What is the ecological implication of the observed differences in the potential response patterns? One possible answer is that the potential responses are the analog of responses to naturally occurring mineral compounds, as there are so many kinds of redox-active minerals (e.g., iron- and manganese-oxides) on Earth. Furthermore, minerals with the same composition but with different crystal phases often have different redox potentials. For example, the redox potential of goethite (α-FeOOH, −0.27 V vs. standard hydrogen electrode (SHE)) is much more negative than that of ferrihydrite (amorphous FeOOH, −0.1 to +0.1 V vs. SHE) [40]. Previous studies have demonstrated that the existence of different iron oxide species results in the dominance of different *Geobacter* species in rice paddy soil [41] and in enrichment cultures from sediments [42]. It has also been reported that electrochemical reactivity with different iron oxide minerals are different among *Geobacter* species [37,43,44]. These observations indicate that the dominance of different *Geobacter* species and/or the switching of EET paths in a single *Geobacter* species would also occur in natural environments depending on the presence of different redox active minerals.

It has been considered that selective enrichment of bacteria with superior current-generating abilities (e.g., *G. sulfurreducens*) on anodic electrodes is a possible strategy in achieving an improvement in the power output of MFCs. However, effective methods for selective enrichment of specific bacteria have not yet been developed. This study points out that the tuning of anode potentials could be a promising method for the selective enrichment of specific current-generating bacteria. However, several previous studies have reported that differences in biofilm biomass on electrodes and anode materials (e.g., graphite vs. ITO electrodes) affect response features of *G. sulfurreducens* to anode potentials [21,27]. Further studies on the effects of anode potentials on various current-generating bacteria including non-model microorganisms would facilitate the development of methods for the selective enrichment of high-output microbial consortia in BES’s.

## 3. Materials and Methods

### 3.1. Bacterial Strains and Culture Conditions

*G. sulfurreducens* (DSM 12127^T^), *G. metallireducens* (DSM 7210^T^), *G. daltonii* (DSM 22248^T^), *G. bemidjensis* (DSM 16622^T^), *G. chapellei* (DSM 13688^T^), and *G. pelophilus* (DSM 12255^T^) were purchased from the Deutsche Sammlung von Mikroorganismen und Zellkukturen GmbH (Braunschweig, Germany). The *Geobacter* species were routinely cultivated in PSN medium [37] with acetate (10 mM) and fumarate (40 mM) as the electron donor and acceptor, respectively, with the exception of nitrate (10 mM) as an electron acceptor for *G. metallireducens*. The *Geobacter* species were cultivated at 30 °C under an atmosphere of N_2_/CO_2_ (80:20 (*v*/*v*)) without shaking.

### 3.2. Electrochemical Analysis

**Figure 3:**
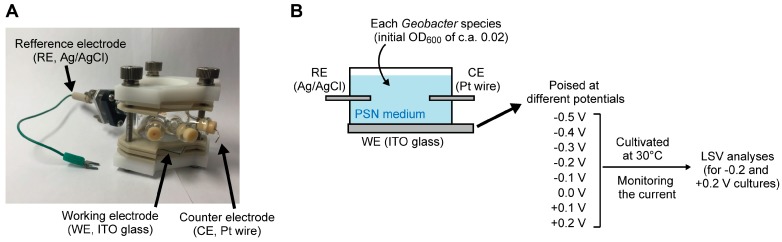
(**A**) The appearance of an electrochemical cell used in this study; (**B**) The experimental scheme of this study.

The experimental scheme for the electrochemical cultivation and following electrochemical analyses were summarized in Figure 3. A single-chamber three-electrode electrochemical cell (14 mL capacity, Figure 3A) with an ITO glass electrode (an effective surface area of 6.2 cm^2^) as the working electrode (anode), located at the bottom of the electrochemical cell, was used. An Ag/AgCl (KCl saturated) electrode and a platinum wire were used as the reference and counter electrodes, respectively. After sterilization, the electrochemical cells were filled with the sterilized PSN medium [32] containing 10 mM of acetate. After purging with N_2_/CO_2_ (80:20 (*v*/*v*)) gas for 5 min to remove dissolved O_2_, 200 µL of pre-cultured *Geobacter* cells (optical densities of ca. 0.3) were inoculated. The working electrode was poised at −0.5 to +0.2 V vs. Ag/AgCl throughout the cultivation using an HA-151B Potentiostat/Galvanostat (Hokuto Denko, Tokyo, Japan, and the current was recorded every 10 min. The electrochemical cultivations were conducted in triplicate.

After the electrochemical cultivation at −0.2 or +0.2 V, each *Geobacter* species was subjected to the LSV analyses when the current productions approached to the respective maximum values. The LSV analyses were performed using an HSV-110 Automatic Polarization System (Hokuto Denko). The parameters for LSV were as follows: equilibrium potential = −0.7 V, equilibrium time = 20 s, scan rate = 5 mV·s^−1^, *E*_initial_ = −0.7 V, and *E*_final_ = +0.3 V. The LSV data were processed using HSV-110 Remote & Analysis Software (Hokuto Denko). The electrochemical analyses were conducted in triplicate, and the representative data are shown in the figures.

## 4. Conclusions

In this study, the variations in the response of diverse *Geobacter* species to changes in anode potentials were investigated for the first time. The electrochemical cultivation clearly demonstrated the differences in the effects of anode potentials on the current generation of *Geobacter* species: the *G. metallireducens* clade species (*G. sulfurreducens* and *G. metallireducens*) constantly generated a large current at a wide range of anode potentials, while the subsurface clades species (*G. daltonii*, *G. bemidjensis*, *G. chapellei*, and *G. pelophilus*) generated a relatively large current only at limited potential regions. The following electrochemical analyses showed that the *G. metallireducens* clade species utilize only one EET path irrespective of anode potentials, while the subsurface clades species utilize multiple EET paths and can switch them depending on the anode potentials. This study reveals that an appropriate tuning of anode potentials would be beneficial for the selective enrichment of specific bacteria and/or control of in situ activities of certain current-generating bacteria.

## Supplementary Materials

Supplementary materials can be found at www.mdpi.com/1422-0067/18/1/108/s1.

## Abbreviations

EET	extracellular electron transfer
OMC	outer membrane *c*-type cytochrome
MFC	microbial fuel cell
BES	bioelectrochemical system
ITO	tin-doped indium oxide
LSV	linear sweep voltammetry
