# Supplementary material for: Influence of Anode Potentials on Current Generation and Extracellular Electron Transfer Paths of Geobacter Species
Source: Int J Mol Sci. 2017 Jan 6;18(1):108. doi: 10.3390/ijms18010108 (PMC5297742; doi:10.3390/ijms18010108)
Supplement: Supplementary file 1 [file ijms-18-00108-s001.pdf]

Int. J. Mol. Sci. **2017**, *18*, 108; doi:10.3390/ijms18010108

Supplementary Materials: Influence of Anode Potentials on Current Generation and Extracellular Electron Transfer Paths of *Geobacter* Species

Souichiro Kato

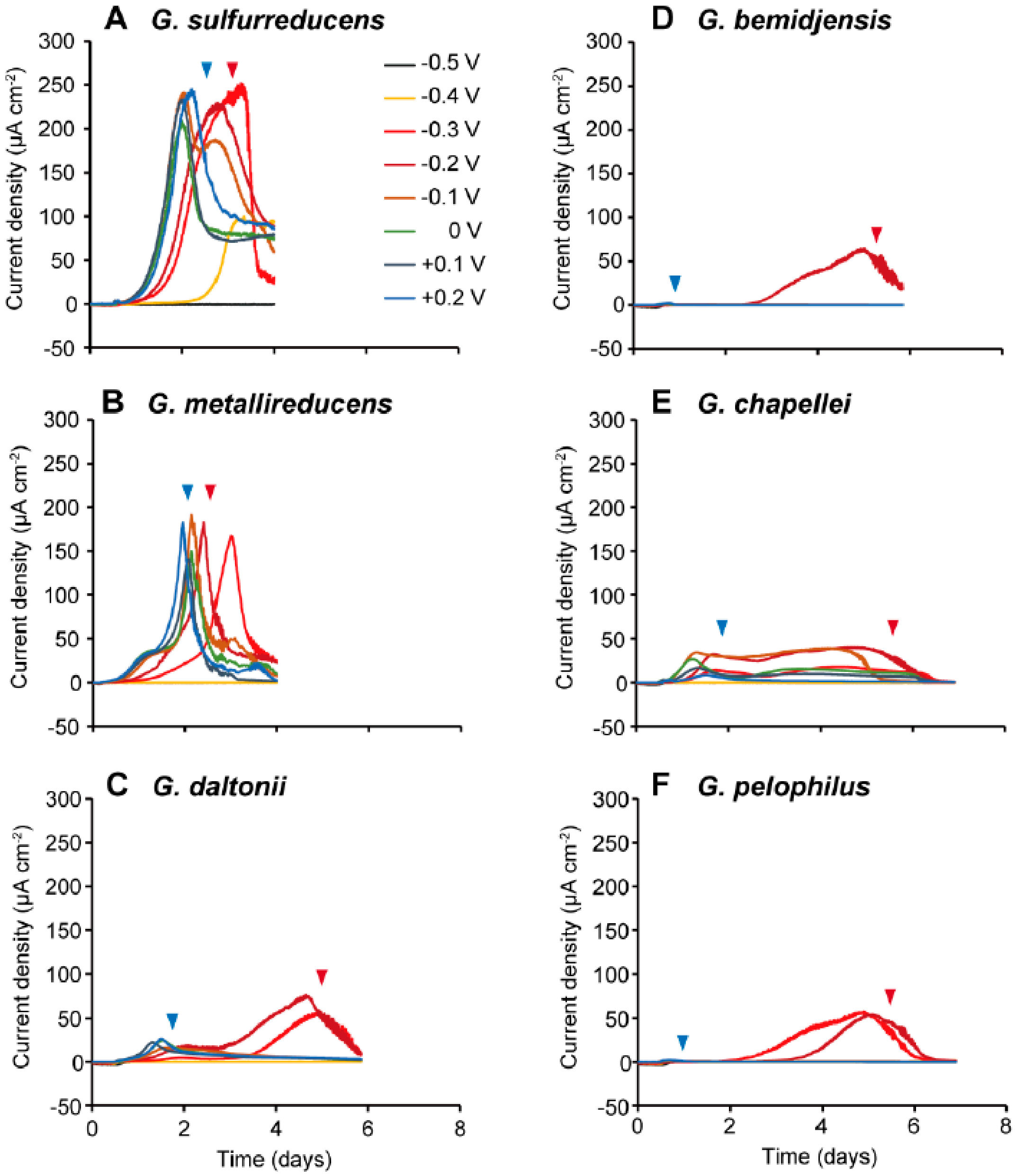

Figure S1. The current–time plots of *Geobacter* species grown with different anode potentials. Red and blue arrowheads represent the time points of linear sweep voltammetry (LSV) analyses for −0.2 and +0.2 V cultures, respectively. The experiments were conducted in triplicate, and the figure shows the representative one.
